# Effectiveness of a 5-day adapted swim instruction program for children with disabilities

**DOI:** 10.3389/fresc.2024.1496185

**Published:** 2025-01-03

**Authors:** Tana B. Carson, J. Megan Irwin, Tania Santiago Perez, Ivana Frampton, Lisa Ruby

**Affiliations:** ^1^ Department of Occupational Therapy, Florida International University, Miami, FL, United States; ^2^ Department of Health and Human Performance, College of Charleston, Charleston, SC, United States; ^3^ iCan Shine, Inc., Miami, FL, United States; ^4^ Department of Counseling, Recreation and School Psychology, Florida International University, Miami, FL, United States

**Keywords:** drowning prevention, water safety, swim instruction, adapted, adaptive, disability

## Abstract

**Background:**

Drowning is a leading cause of death for children. Some populations of children with disabilities, such as children with autism, experience a health disparity in drowning when compared to peers without disabilities.

**Objective:**

This study presents a secondary data analysis of the response to intervention for a 5-day adapted swim instruction program (iCan Swim) for children with disabilities (*n* = 164 participants) ages 3–18 years.

**Methods:**

This secondary data analysis assessed the effectiveness of the swim intervention on changes in swim skill level from Day 1 to Day 5. Associations between response to intervention (i.e., change score) and participant characteristics were examined using Kendall's tau-b for age and Chi-square for sex and diagnosis. Models were fit using a Poisson regression to examine potential predictors of progress across participants.

**Results:**

Swim skills significantly improved from Day 1 (*Md* = 1.00, *n* = 164) to Day 5 (*Md* = 2.00, *n* = 164), z = −10.06, *p* < .001, r = .58). Most participants (61.6%) improved by at least one swim skill level. Age was weakly, yet significantly positively correlated with swim skill level change scores (*τ*_b_ = .154, *p* = .020) and was a significant predictor of swim skill level change for participants with Down Syndrome [b = .091, S.E. = .0434, *p* = .036, 95%CI (.006,.176)].

**Conclusions:**

While this 5-day adapted swim instruction program was effective for most participants in improving swim skills, certain factors may have contributed to slower progression including participant fearfulness or needing more time. Further study of these factors is warranted.

## Introduction

Fatal and non-fatal drowning of children is a preventable major public health concern [(1), p. e008444]. In the United States, drowning is the leading cause of death among children ages 1–4, and the second leading cause of death among children ages 5–14 (2). The risk of death by drowning is higher for some disability groups when compared to those without disabilities. For example, the risk of drowning is twice as high for those with autism spectrum disorder (autism) compared to those without autism [(3), no. 4]. According to the National Autism Association “drowning is the leading cause of death among individuals with autism” [(4–6), p. 32 (7), pp. 869–874]. While it is unclear whether the risk of drowning is also greater for those with other developmental disabilities or physical disabilities, it is clear there are barriers to accessing swim instruction services designed to prevent drowning for these populations. Barriers include a lack of access to specialized training for instructors, access to specialized programming for swimmers, or environmental/contextual factors, such as training [(8), pp. 197–207 (9), pp. 419–425 (10), pp. 29–40 (11), pp. 451–455 (12), p. 1179556519872214]. Thus, there is a critical need for improved access to evidence-based drowning prevention interventions for all children, particularly for those with disabilities.

Drowning is a complex problem and drowning prevention interventions may address multiple factors including education for parents, swimming lessons, water safety education, aquatic rescue competency [(13), pp. 688–693] and barriers such as pool fencing [(14), pp. 195–204]. Swim instruction programs are an important part of the solution for drowning prevention (15). Fundamental swimming skills taught in swim instruction (LTS) programs often include the following: safe water entry and exit, floating, breath control, resurfacing after fully submerging, and swimming following submersion, swimming comfortably underwater, and becoming proficient in at least one prone and one supine stroke [(16), no. 4 (17), no. 2].

While community-based LTS programs are readily available across the U.S, through a variety of associations and organizations, children with disabilities are often underrepresented in these programs. Solish and colleagues [(18), pp. 226–236] found that 62.2% of parents of children who do not have disabilities reported participating in swimming lessons, while only 27.7% of parents of children with autism and 43.3% of parents of children with intellectual disability (ID) reported that their children participated in swimming lessons. Participation rates are also lower among ethnic minorities in low socioeconomic areas [(19), pp. 19–33 (20), no. 2]. Thus, inequities experienced by children with disabilities may be further compounded by the intersection of disability and racial/ethnic and/or socioeconomic factors.

To meet the unique needs of individuals with disabilities, adapted sports and recreation programs have been developed and implemented in various settings (e.g., schools, athletic programs, community parks and pools, etc). These programs may modify rules, equipment, facilities and/or the environment to afford people with disabilities access to meaningful and successful participation (21). Adapted aquatics programs serve individuals with disabilities using swimming, aquatic recreational activities, and water safety to promote wellness, habilitation, and rehabilitation [(22), p. 18]. A modified swimming program for individuals with disabilities requires diverse adaptations such as various teaching techniques, modified equipment, and well qualified instructors (23). Within this framework, adapted swim instruction programs have been developed, but few have been studied for their effectiveness.

One adapted program with national reach in the US is the iCan Shine, iCan Swim Camp. iCan Shine is a non-profit organization 501(c)3 “whose mission is to provide quality learning opportunities in recreational activities for individuals with disabilities” (24). iCan Swim is a 5-day adapted swim camp for children with disabilities agedthree years or older that provides instruction of basic swimming and water safety skills. The iCan Swim program curriculum is focused on developing swimming skills that fall within the categories described by the American Red Cross swim instruction program Levels 1–3 (25). All lessons include an emphasis on comfort in water and skills related to safety such as: recognizing lifeguards, entering/exiting in shallow and deep water safely, using a lifejacket, breath control as well as swimming skills such as body positioning and stroke mechanics. When appropriate, advanced swimmers are provided with additional instruction focused on more advanced swimming strokes such as breaststroke and butterfly. Program eligibility criteria include being at least 3 years of age and having a medical, neurological, developmental, or physical diagnosis that is considered a disability. Individuals that have a gastrostomy-tube that is less than two months old, a tracheostomy,or exhibit aggressive behaviours (at the discretion of program directors and instructors) are excluded for safety concerns. Participants attend one swim session daily Monday – Friday for one week. Swimmers are grouped into sessions by age, rather than skill level as accurate skill level may be unknown and to promote developmentally appropriate social groupings (i.e., not have very young children and adolescents in the same group). Each session includes two iCan Shine adapted swim instructors and three to six swimmers who are paired with at least one volunteer swim buddy, one to two additional volunteers may provide general support for the group. Swim sessions last 45-minutes for swimmers aged 3–7 years and 60-minutes for swimmers 8 years and older. The iCan Shine instructors conduct a swim assessment on Day1 to determine participants' current swimming ability and help develop individual goals for their time at camp. Swimmers' skills are reassessed on Day 5 to evaluate and document progress. The typical iCan Swim lesson sequence consists of a water safety lesson, a group game for warm-up, individual or small group skill practice, a group activity for skill reinforcement, and a group activity for wrap-up. A summary of daily training and swim instruction content is provided in the Supplementary Table S1. Since the sequence of skills presented in traditional swim instruction program may not be developmentally valid or appropriate for those with disabilities [(26), pp. 269–285], the adapted framework, as adopted by iCan Swim, allows for an individualized approach where swimmers can work on skills within any level as appropriate for developing functional swimming skills. Additionally, iCan Swim incorporates programmatic factors that have been shown to foster success in adapted swimming programs such as providing training to qualified instructors and 1:1 support for swimmers [(27), p. 1473328, (28)].

Effectiveness of the iCan Swim program has been partially examined. Rogers et al. (23), evaluated the effectiveness of this program for children on the autism spectrum. Their secondary data analysis showed that autistic children demonstrated improvements in performance over the 5-day program. The study also examined factors linked to limitations in performance outcomes such as age and time out of water. Questions remain regarding outcomes for children with other disabilities, whether the same personal factors influence outcomes for other diagnoses and the effectiveness of the 5-day program when accounting for participation (i.e., time in session).

The primary aim of this study is to expound upon the work of Rogers et al. (23) and evaluate the changes observed from Day 1 to Day 5 of iCan Shine's 5-day iCan Swim camp for individuals from various disability groups. Secondary aims explored (a) relationships between intervention response and personal factors (e.g., fearfulness and challenging behaviours) and (b) predictors for change in swim level performance.

## Methods

This study presents a secondary data analysis to determine the effectiveness of a 5-day adapted swim instruction program, iCan Swim, on the development of swimming skills among children with disabilities ages 3 to 18 years. This study was reviewed by Florida International University's Institutional Review Board (IRB) and determined to be exempt (IRB Protocol Non-Human Subjects Research #: IRB-21-0465).

### Secondary data

Can Shine provided the research team with deidentified data from the nine iCan Swim camps conducted throughout the US between April and August of 2019. The database contains demographic data (e.g., age, diagnosis) obtained from caregiver-reported registration documents and data from the swim skill assessments conducted by the iCan Shine swim staff.

### Sample

The complete dataset provided by iCan Shine included 234 participants. Participants were included in our analysis if they met the following criteria: (1) were between the ages of 3–18 years, (2) had at least 1 formal diagnosis, (3) had beginning (Day 1) and ending (Day 5) swim skill data, and (4) did not miss more than 50-minutes of swim instruction (i.e., the equivalent of one session) over the five days. The sample selection process is presented in Supplementary Figure S1.

### Demographics

Online registration forms were used to collect pertinent information on iCan Swim program participants' demographics. Information extracted included participants' medical diagnoses, age, sex, and gender. Parents were able to report up to four diagnoses on the registration form. Participants were grouped for data analyses as follows using the primary reported diagnosis: Down Syndrome (DS), autism (ASD), or other.

### Swim skill assessments

Swim skills were assessed by the iCan Swim instructors using an assessment tool developed by the iCan Shine staff for the program that is based on the American Red Cross swim levels 1–3 (25). Following the skills assessment, the swim instructors assigned swimmers into the following swim levels based on their assessment performance: non-swimmer (NS,), beginner swimmer (BS), intermediate swimmer (IS) or advanced swimmer (AS,). See Table 1 for operational definitions of each swim skill level. Swim skill levels reported for Day 1 (starting level) and Day 5 (ending level) were used to determine change in swim skill level across the camp (i.e., intervention). For participants that did not progress by at least one swim level by Day 5, iCan Swim instructors were asked to identify one of the following reasons for lack of advancement: (1) need for more practice, (2) fearfulness, (3) the swimmer displayed challenging behaviors that impeded progress, (4) readiness (i.e., developmentally), or (5) fitness.

**Table 1:** iCan Swim skill level definitions.

Swim skill level	Abbreviation	Definition
Non-swimmer	NS	Refuses to get in pool or refuses to try most skills once in the pool. Unable to be independent on a noodle.
Beginning swimmer	BS	Independent and trying skills on a noodle; unable to swim 2 body lengths of any stroke independently without a noodle.
Independent swimmer	IS	Able to swim any stroke 2 body lengths without noodle; able to jump in, recover, and get to the wall.
Advanced swimmer	AS	Has mastered basic skills and is usually at camp to work on stroke refinement (e.g., rotary breathing, competitive strokes) or swim endurance (i.e., swim more than 1 lap).

### Response to intervention

The research team created the variable “change score” to represent the change in a participant's swim level from Day 1 to Day 5. Scores ranged from 0-no change in swim skill level to 3-swim skill level increased by three levels.

### Data analysis

All statistical analyses were conducted using IBM SPSS Statistics (Version 27). Examination for outliers identified participants (*n* = 2) whose swim skill level on Day 1 was in the interquartile range of 1.5. These participants demonstrated the highest swim skill level (i.e., Advanced Swimmer) and were subsequently removed from further data analyses as they demonstrated a ceiling effect on the swim assessment. Assumptions of normality assessed using Kolmogorov-Smirnov tests revealed that the data were not normally distributed. Therefore, a Wilcoxon Signed Rank test was used to assess program effectiveness (i.e., improvement in swim skill level from Day 1 to Day 5). Associations between response to intervention (i.e., change score) and participant characteristics were examined using Kendall's Tau-b for age and Chi-square for sex and diagnosis. For all analyses, age was categorized as: early childhood (ages 3 - < 6 years), middle childhood (6 - < 10years), late childhood (10 - < 13), or adolescence (13–18 years). Models were fit using a Poisson regression to examine potential predictors of progress across campers.

## Results

### Demographics

A total of 164 participants (113 males, 51 females, M_age_ = 9.14 ± 3.53 years) were included in the data analyses. Autism comprised the largest diagnostic group (*n* = 72, 43.9%), followed by DS (*n* = 57, 34.8%), and then Other (*n* = 35, 21.3%). Table 2 provides additional information regarding the sample distribution based on sex and age.

**Table 2:** Participant demographics by diagnosi*s.*

		Sex *f* (%)		Age (years) *f* (%)			
Group	*f*	Male	Female	Early childhood (3 - < 6)	Middle childhood (6 - < 10)	Late childhood (10 - < 13)	Adolescence (13–18)
Total	164	113 (68.9)	51 (31.1)	30 (18.3)	57 (34.8)	47 (28.7)	30 (18.3)
Autism	72	63 (87.5)	9 (12.5)	15 (20.8)	22 (30.6)	21 (29.2)	14 (19.4)
DS	57	32 (56.1)	25 (43.9)	12 (21.1)	19 (33.3)	18 (31.6)	8 (14.0)
Other	35	18 (51.4)	17 (48.6)	3 (8.6)	16 (45.7)	8 (22.9)	8 (22.9)

DS, down syndrome.

### Swimming skill level

More than half of all participants started at a swim skill level classification of non-swimmer (NS, 53%). By Day 5, the NS group was reduced by more than half to 22.5% of the total sample. Overall, 63.4% of the total sample demonstrated measurable swim skill improvements by advancing one to two swim skill levels by Day 5. From Day 1 to Day 5, most participants (61.6%) progressed by at least one swim skill level, some (36.6%) maintained the same skill level, and few (1.8%) progressed two swim skill levels (see Table 3). No participants advanced three swim skill levels in the 5-day program. A Wilcoxon signed rank test confirmed statistically significant improvement in the overall sample from Day 1 (*Md* = 1.00, *n* = 164) to Day 5 (*Md* = 2.0, *n* = 164), z = - 10.06, *p* < .001, with a strong effect size, r = .58. Level of change scores are presented in Table 3, while level of progression by diagnosis and start level are presented in Table 3.

**Table 3:** Swim skill improvement organized by start vs. end level and by diagnosis.

Start level		End level			
	*n*	Non-swimmer (NS)	Beginner swimmer (BS)	Intermediate swimmer (IS)	Advanced (AS)
Non-swimmer		*f* (%)	*f* (%)	*f* (%)	*f* (%)
Total	87	37 (42.5)	48 (55.2)	2 (2.3)	–
Autism	34	13 (38.2)	21 (61.8)	–	–
DS	32	20 (62.5)	10 (31.3)	2 (2.3)	–
Other	21	4 (19.0)	17 (81.0)	–	–
Beginning swimmer					
Total	64	–	20 (31.3)	43 (67.2)	1 (1.6)
Autism	32	–	13 (40.6)	18 (56.3)	1 (3.1)
DS	23	–	4 (17.4)	19 (82.6)	–
Other	9	–	3 (33.3)	6 (66.6)	–
Intermediate swimmer					
Total	13	–	–	3 (23.1)	10 (76.9)
Autism	6	–	–	–	6 (100)
DS	2	–	–	1 (50)	1 (50)
Other	5	–	–	2 (40)	3 (60)
		Level of Improvement			
		No change	Improved 1 level	Improved 2 levels	Improved 3 levels
Diagnosis		*f* (%)	*f* (%)	*f* (%)	*f* (%)
Totals	164	60 (36.6)	101 (61.6)	3 (1.8)	–
Autism	72	26 (36.1)	45 (62.5)	1 (1.4)	–
DS	57	25 (43.9)	30 (52.6)	2 (3.5)	–
Other	35	9 (25.7)	26 (74.3)	–	–

### Reasons for not progressing

When participants did not progress at least one swim skill level, instructors from iCan Swim noted reasons for the lack of swim skill progression. The distribution of reasons for not progressing are summarized according to diagnostic category and by start level (Table 4). According to diagnosis grouping, the most reported reason for not progressing at least one level was “fearfulness” for swimmers with ASD *n* = 11 (42.3%) and “needs more practice” for swimmers with DS *n* = 12 (48%; see Table 4). According to starting level groups, the most reported reason participants did not progress at least one level was “fearfulness” for non-swimmers *n* = 18 (48.6%) and “needs more practice” for the beginning swimmers *n* = 13 (65%; Table 4). Behavior was only listed as a reason for not progressing for one participant in the intermediate swim group.

**Table 4:** Instructor rationale for those with no change in swim level: organized according to diagnosis or start level.

		Rationale					
		No rationale reported	Needs more practice	Fearfulness	Readiness	Mobility or endurance	Behavior
Diagnosis	*n*	*f* (%)	*f* (%)	*f* (%)	*f* (%)	*f* (%)	*f* (%)
Total	60	8 (13.3)	24 (40.0)	21 (35.0)	4 (6.6)	2 (3.3)	1 (1.6)
Autism	26	5 (19.2)	10 (38.4)	11 (42.3)	–	–	–
DS	25	1 (4.0)	12 (48.0)	7 (28.0)	4 (7.0)	–	1 (1.8)
Other	9	2 (22.2)	2 (22.2)	3 (33.3)	–	2 (22.2)	–
		No Rationale Reported	Needs More Practice	Fearfulness	Age	Mobility or Endurance	Behavior
Start level	*n*	*f* (%)	*f* (%)	*f* (%)	*f* (%)	*f* (%)	*f* (%)
Non-swimmer							
Total	37	3 (8.1)	11 (29.7)	18 (48.6)	4 (10.8)	1 (2.2)	–
Autism	13	2 (15.3)	2 (15.3)	9 (69.2)	–	**–**	–
DS	20	1 (5.0)	9 (45.0)	6 (30.0)	4 (20.0)	–	–
Other	4	–	–	3 (75.0)	–	1 (25.0)	–
Beginner swimmer							
Total	20	3 (15.0)	13 (65.0)	3 (15.0)	–	1 (5.0)	–
Autism	13	3 (23.1)	8 (61.5)	2 (15.3)	–	–	–
DS	4	–	3 (75.0)	1 (25.0)	–	–	–
Other	3	–	2 (66.6)	–	–	1 (33.3)	–
Intermediate swimmer							
Total	3	2 (66.6)	–	–	–	–	1 (33.3)
Autism	–	–	–	–	–	–	–
DS	1	–	–	–	–	–	1 (100)
Other	2	2 (100)	–	–	–	–	–

### Predictors of change in swim skill level

A Kolmogorov-Smirnov test indicates that change in swim skill level values did not follow a normal distribution, D(164) = .384, *p* < .001. Therefore, Kendall's Tau-b associations between response to intervention (i.e., change score) and participant characteristics were examined for age, and Chi-square associations were utilized for sex and diagnosis. Results revealed that age had a weak yet significant positive relationship with change in swim skill level (*τ*_b_ = .154, *p* = .020) and neither sex (*χ*^2^_(2, *N*_ _=_ _164)_ = 1.872, *p* = .392) nor diagnosis *χ*^2^_(2,*N*_ _=_ _164)_ = 5.223, *p* = .265) were significantly related to change in swim skill level. Based on these results, Poisson regressions were modeled to examine age as a predictor of change in swim skill level.

The first Poisson regression examined age as a predictor of change in swim skill level for the whole sample. The likelihood ratio chi-square test indicated that the full model (age as a predictor) was not a significant improvement of the null (intercept only) model (*p* = .126). Accordingly, age was not a significant predictor of change in swim skill level from Day 1 to Day 5 (b-.042, S.E. = .0271, *p* = .124). Due to concerns that any effect of age might be masked when comparing children with different disabilities together, the data were split by diagnosis group and a model was fit for each group. For these models, the likelihood ratio chi-square test indicated that the full mode was a significant improvement in fit over the null (intercept only) model for the DS group (*p* = .039) but not for the autism (*p* = .724) or Other disabilities (*p* = .942) groups. For individuals with DS, age was a significant predictor of change in swim skill level [b = .091, S.E. = .0434, *p* = .036, 95%CI (.006,.176)]. The incidence rate ratio indicated that for each year increase in age, the incidence rate (for change in swim skill level) increased by a factor of 1.095, or 9.5%.

## Discussion

The purpose of this study was to evaluate the effectiveness of a 5-day adapted swim instruction program, iCan Swim, on the progression of swim skills (according to predefined skill levels) in a sample of 164 children with disabilities ages 3 to 17 years. Consistent with a previous study determining the efficacy of iCan Swim programs on children with autism [(23), p. 5557], most participants (61.6%) in the present study improved their swim skill level from Day 1 to Day 5, regardless of age, sex, or diagnosis. These results suggest that children with disabilities can acquire swim skills in a brief time limit (5 days). Having participants progress from non-swimmers to swimmers in a brief time limit demonstrates the feasibility of rapid skill acquisition when appropriate supports and instruction are provided to people with disabilities.

The peer-reviewed literature presents variability, and it seems there is little consensus on what presents a valid, effective adapted swim instruction curriculum for children with disabilities, along with best practices for program development and implementation. Forde et al. (17) reported on an inclusive program for children with and without disabilities that focused on six swimming and water safety skills during 10, 30-min sessions over a two-week period; the six skills targeted included (1) not swimming alone, calling for help and reaching and throwing when others are in distress, (2) entering the water by jumping in, (3) performance of progressive arm stroke, (4) performance of back float with no support for three seconds, (5) skill in jumping in, turning, and stoke/kick to the wall, and (6) skill to exit the water using ladder, step or side. Jorgic et al. (29) utilized the Halliwick Method along with swimming exercises and measured the effects of this swimming program on the gross motor function, mental adjustment to the aquatic environment, and the ability to move and swim in the water among 7 children with cerebral palsy; the intervention consisted of 12, 45-minute sessions over six weeks. Lawson et al. (30) investigated the effects of KU Sensory Enhanced Aquatics program, an individualized swim instruction program that used sensory preferences and task analysis to teach swimming to children with autism (*n* = 83); the program consisted of 8, 30-minute sessions over 8 weeks and the swim skills measured included water orientation, front stroke, backstroke, breaststroke, butterfly, diving, and use of googles. Levy et al. (31) investigated the effectiveness of a behavioral treatment package of shaping, prompting, and positive reinforcement to teach underwater submersion to children with autism (*n* = 3); each session lasted 30-minutes, with the length of the intervention ranging from 12 to 27 sessions among the 3 participants. Kemp et al. (27) examined the efficacy of AquOTic, an occupational-therapy-based aquatic intervention (*n* = 37) that includes play and child-based activities, task-specific training, positive reinforcement, sensory supports, and a modified Halliwick approach; the program consisted of ten 60-min sessions over 10 weeks. Rogers et al. (23) utilized the same iCanSwim program as the present study, but they only included children with autism in their analysis. Compared to existing programs iCan Swim is on the higher end of duration (45–60-min/session) and the lower end of the frequency of sessions (5 sessions).

Age had a weak but positive relationship with changes in swim skill acquisition overall. Further analysis revealed that this correlation was primarily driven by the DS group. Within the DS group, as age increased each year, swim skill acquisition increased by 9.5%. Age was not correlated with changes in swim acquisition for children with autism; these results did not replicate other research findings that suggested age-related differences in children with autism [(23), p. 5557]. It is important to consider that the present study purposefully screened out factors such as time out of water and behavioral issues as exclusion criteria, which may account for why age was not related to swim skill acquisition in the autism group in this study.

iCan Swim is typically led by two skilled swim instructors with training in adapted aquatics. Swimming is a complex motor skill and teaching swimming and water safety to children with disabilities requires training, expertise, and a strong knowledge base of disability and diagnosis specific instructional strategies. While most aquatic professionals and staff are well trained in water safety and swim instruction instruction, many report a lack of training on disability and the appropriate modifications or accommodations needed to teach swimming and water safety to children with disabilities (10). Traditional swim instruction programs are effective for children without disabilities. However, they may not be developmentally valid for those with physical or developmental disabilities (26). Thus, it is reasonable to suspect that training in adapted swim instruction vs. basic swim instruction training may have impacts on the speed of swim skill acquisition for children with disabilities. Future work with superiority trials comparing programs that differ in instructor training and other programming factors are needed to better understand which methods are superior. Overall, increased availability of inclusive or adapted swimming programs are warranted to promote the acquisition of safety and swimming skills for children with disabilities.

Strengths of this study include the large sample size and the inclusion of swimmers with disabilities other than autism, of which most research has been focused recently. Having an expanded sample exposed to the same program allows for a preliminary look at potential differences in progress among groups. For those that did not progress to a higher swim skill level, swim instructors noted reasons for not progressing when a clear reason was available. For children with autism, the main reason reported was fearfulness, while for children with DS, the main reason reported was the need for more instruction time. These results suggest that children with autism and children with DS have unique needs that may require different instruction strategies, adaptations, and programmatic considerations. Thus, we recommend additional research to identify best-practices for meeting diagnostic-specific needs (e.g., DS or autism) or characteristic-specific needs (e.g., anxiety, rate of learning, behavioral difficulties, etc.). Researchers and providers of adapted swim instruction programs should carefully evaluate individual swimmer characteristics and program factors to determine best practices for program delivery. A systematic review of evidence-based practices is warranted to better understand the optimal approach, frequency, duration, and participant-treatment matching for adapted swim instruction.

### Limitations

This study employed a secondary data analysis based on a limited dataset. The data used for the study were collected from a community-based program. Inter-rater reliability of the assessment process has not been evaluated for this dataset. Additionally, this study was limited to evaluating swim progression based on swim levels rather than discrete skill development. For example, while some participants may not have progressed a full level, they may have demonstrated measurable improvements in important skills within levels such as breath control, increased comfort in the water, etc. These benefits are important and can lead to further success in ongoing swim lessons beyond the 5-day program but were not measured in the current study. Additional factors could reasonably be expected to impact success such as cognition, comfort in water, mobility, attention, diagnostic complexity (i.e., number of diagnoses and severity) but this information was not available for the current study. Although iCan Swim includes a water safety education component, outcomes of water safety education were not available in this limited dataset. We recommend future studies evaluating outcomes of water safety education in addition to swimming skills (e.g., identifying the lifeguard and use of safety equipment such as life jackets). Participants of the program may have demonstrated improvements in these skills, which were not captured by the swim skill level assessments. Part of the inclusion criteria for this secondary data analysis was missed lesson time. If a participant missed more than 50-min of their session, their data was not included. For future studies lesson time missed could be calculated and explored as a predictive variable for success. Missed lesson time could be due to anything from late arrival, early departure, to missing an entire session or time out of water due to behavior issues. Each of these factors could have unique impacts on swim skill development. Because time out of water was recorded, but reasons for time out of water were not recorded, these factors and their relationship to swim skill development could not be addressed in the present study.

### Future research

Teaching basic swimming and water safety skills to school-age children is one of three global interventions to prevent drowning proposed by the World Health Organization (32). The other two interventions are the provision of community-based supervised daycare for pre-school children, and the provision of training to bystanders on safe rescue and resuscitation. A systematic review of implementation strategies for drowning prevention in high-income countries (*n* = 49) revealed that peer-reviewed drowning prevention interventions mostly include pool-based swimming/water safety lessons, as well as training for parents, followed by beach safety education, and cardiopulmonary resuscitation first aid or bystander training. Other interventions less frequently studied include life jacket use and pool fencing [(33), p. 45]. To our knowledge, no systematic reviews have been published comparing the effectiveness of different drowning prevention interventions for children with disabilities. Authors recommend future studies explore additional drowning prevention interventions such as training for parents, fencing, and bystander training.

In our assessment of the existing literature, it seems there is little consensus on what presents a valid, effective adapted swim instruction curriculum for children with disabilities. Additional research is needed to determine the most effective programmatic factors (e.g., frequency, duration, and approach), and participant factors (e.g., age, diagnosis, fearfulness, etc.) that may influence progress [(12), p. 1179556519872214]. Determining intervention effectiveness also requires a working definition of water competency for this population. While the American Red Cross has identified five critical skills required for water safety/competency, the content validity of these skills has not been evaluated among children with disabilities. Further defining water competency skills for people with disabilities will help improve our ability to match participants to the types of services that are optimal for them to achieve developmentally appropriate levels of water competency.

## Conclusion

The iCan Swim 5-day adapted swim instruction program was effective for most participants. It is, however, important to note that this program was led by skilled instructors with adapted aquatics training. Predictive factors such as fearfulness, needing more time and age should be further studied to better understand these factors and how to develop strategies for overcoming them. In our study, age was a significant predictor of swim skill acquisition within the DS group only. Primary reasons for not progressing to a higher swim skill level were: (1) fearfulness (most often among participants with autism) and (2) more practice needed (most often among participants with DS). Additional factors should be further explored in future research to better understand optimal participant-treatment matching for frequency/duration of lessons. Continuous efforts in the development and evaluation of evidence-based best practices as to when and how to deliver adapted swim instruction to children with disabilities are needed.

## Data Availability

The data analyzed in this study is subject to the following licenses/restrictions: For information regarding the availability of this dataset, contact Lisa Ruby at iCan Shine, Inc. Requests to access these datasets should be directed to lisa@icanshine.org.
