# Supplementary figures and images for: Effectiveness of a 5-day adapted swim instruction program for children with disabilities
Source: Front Rehabil Sci. 2025 Jan 3;5:1496185. doi: 10.3389/fresc.2024.1496185 (PMC11738942; doi:10.3389/fresc.2024.1496185)

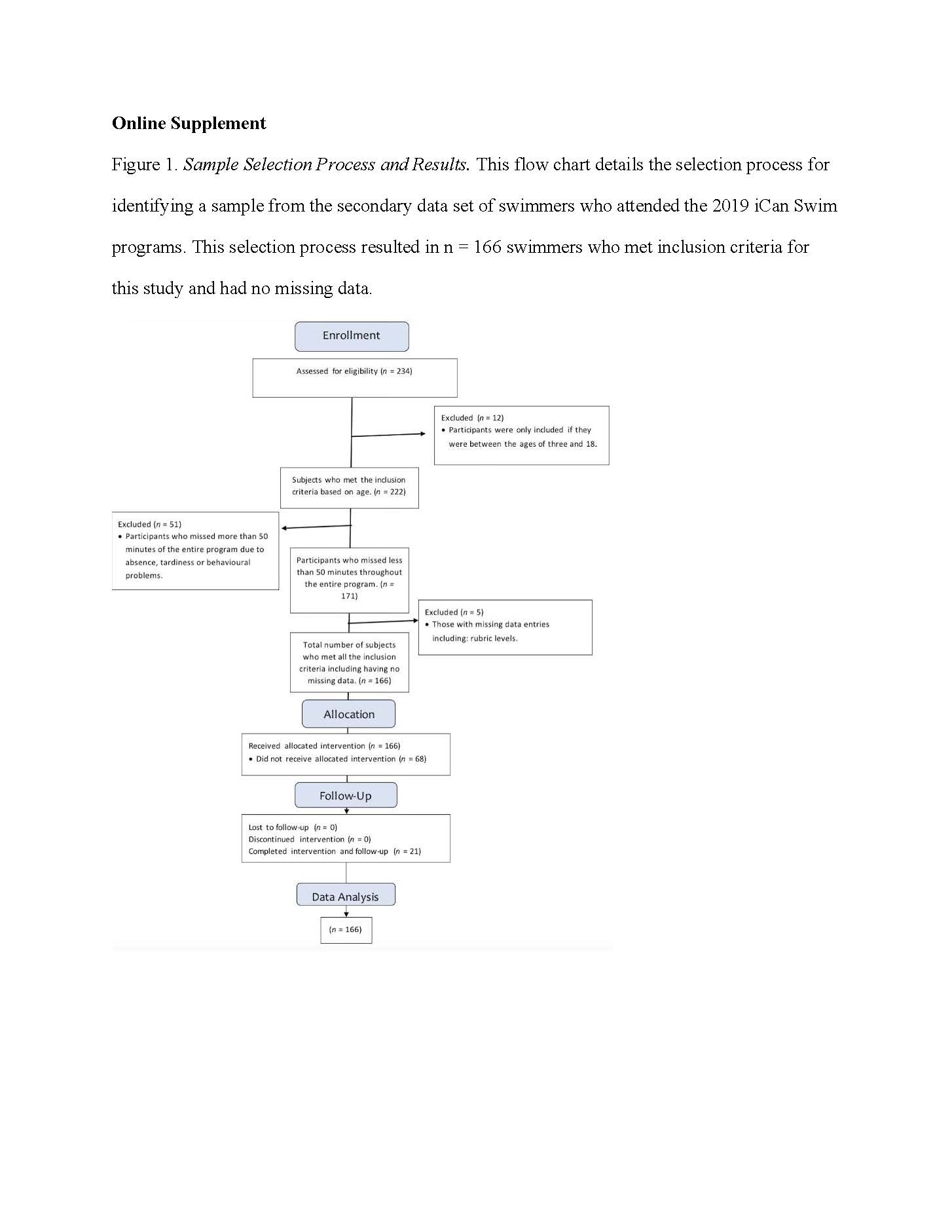

Supplement: Supplementary file 1 [file Image1.jpeg]

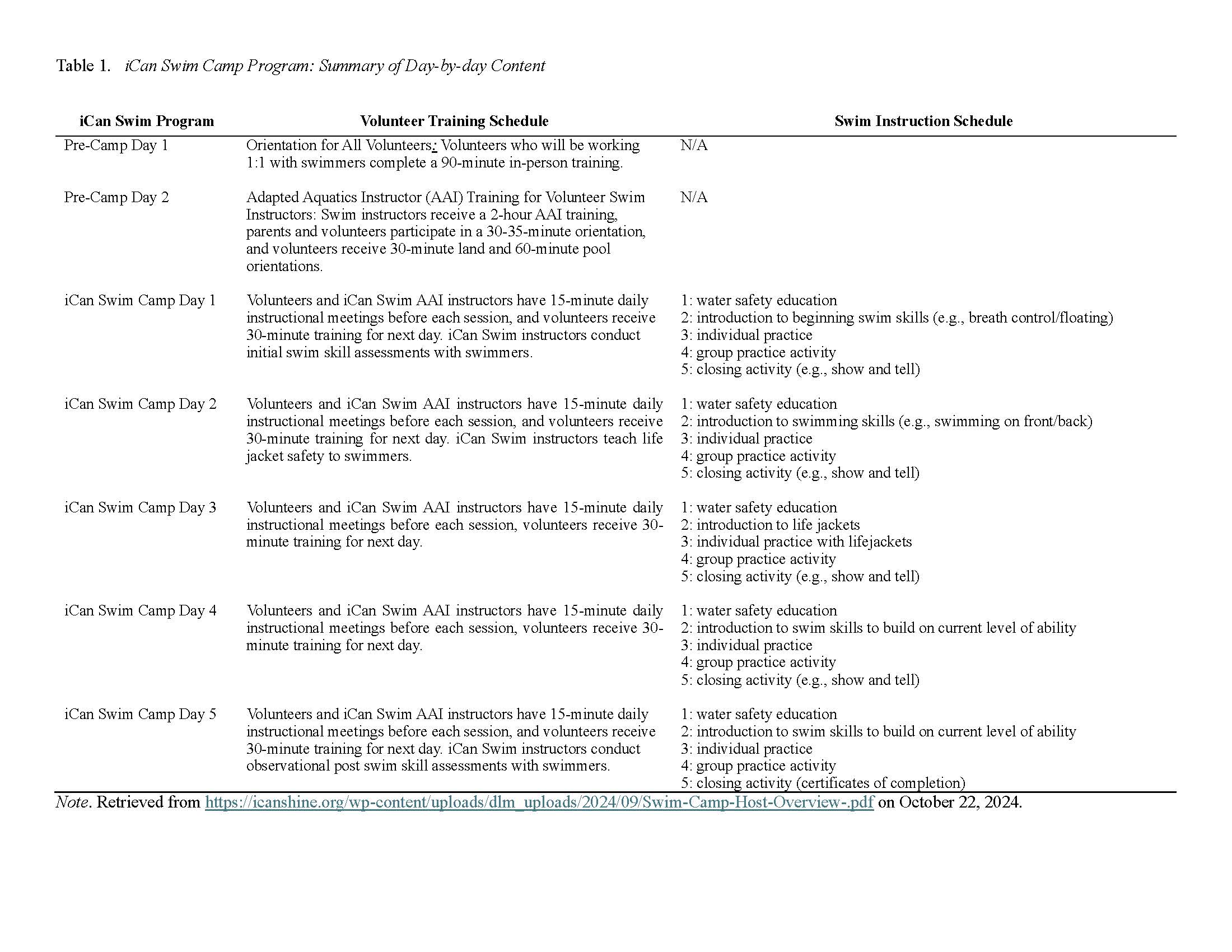

Supplement: Supplementary file 2 [file Supplementaryfile1.jpeg]
